# First-in-human study of a novel cell death tracer [^99m^Tc]Tc-Duramycin: safety, biodistribution and radiation dosimetry in healthy volunteers

**DOI:** 10.1186/s41181-023-00207-1

**Published:** 2023-08-30

**Authors:** Taco Metelerkamp Cappenberg, Stijn De Schepper, Christel Vangestel, Stef De Lombaerde, Leonie wyffels, Tim Van den Wyngaert, Jeffrey Mattis, Brian Gray, Koon Pak, Sigrid Stroobants, Filipe Elvas

**Affiliations:** 1 Department of Nuclear Medicine, Antwerp University Hospital (UZA), Edegem, Belgium; 2 Molecular Imaging and Radiology (MIRA), University of Antwerp, Wilrijk, Belgium; 3 Molecular Targeting Technologies, Inc., West Chester, PA USA

**Keywords:** Cell death imaging, Apoptosis, ^99m^Tc-duramycin SPECT, Biodistribution, Internal dosimetry

## Abstract

**Background:**

Imaging of cell death can provide an early indication of treatment response in cancer. [^99m^Tc]Tc-Duramycin is a small-peptide SPECT tracer that recognizes both apoptotic and necrotic cells by binding to phosphatidylethanolamine present in the cell membrane. Preclinically, this tracer has shown to have favorable pharmacokinetics and selective tumor accumulation early after the onset of anticancer therapy. In this first-in-human study, we report the safety, biodistribution and internal radiation dosimetry of [^99m^Tc]Tc-Duramycin in healthy human volunteers.

**Results:**

Six healthy volunteers (3 males, 3 females) were injected intravenously with [^99m^Tc]Tc-Duramycin (dose: 6 MBq/kg; 473 ± 36 MBq). [^99m^Tc]Tc-Duramycin was well tolerated in all subjects, with no serious adverse events reported. Following injection, a 30-min dynamic planar imaging of the abdomen was performed, and whole-body (WB) planar scans were acquired at 1, 2, 3, 6 and 23 h post-injection (PI), with SPECT acquisitions after each WB scan and one low-dose CT after the first SPECT. In vivo ^99m^Tc activities were determined from semi-quantitative analysis of the images, and time-activity curves were generated. Residence times were calculated from the dynamic and WB planar scans. The mean effective dose was 7.61 ± 0.75 µSv/MBq, with the kidneys receiving the highest absorbed dose (planar analysis: 43.82 ± 4.07 µGy/MBq, SPECT analysis: 19.72 ± 3.42 μGy/MBq), followed by liver and spleen. The median effective dose was 3.61 mSv (range, 2.85–4.14). The tracer cleared slowly from the blood (effective half-life of 2.0 ± 0.4 h) due to high plasma protein binding with < 5% free tracer 3 h PI. Excretion was almost exclusively renal.

**Conclusion:**

[^99m^Tc]Tc-Duramycin demonstrated acceptable dosimetry (< 5 mSv) and a favorable safety profile. Due to slow blood clearance, optimal target-to-background ratios are expected 5 h PI. These data support the further assessment of [^99m^Tc]Tc-Duramycin for clinical treatment response evaluation.

*Trial registration*: NCT05177640, Registered April 30, 2021, https://clinicaltrials.gov/study/NCT05177640.

**Supplementary Information:**

The online version contains supplementary material available at 10.1186/s41181-023-00207-1.

## Background

In the era of expanding therapeutic options and personalized medicine, early monitoring of tumor response to therapy has gained significant importance. The ability to detect changes at the molecular level before they become apparent morphologically has made molecular imaging techniques like positron emission tomography (PET) and single-photon emission computed tomography (SPECT) instrumental for evaluating treatment response at an early stage. These techniques can provide higher sensitivity and specificity in differentiating early responders from non-responders after cancer treatment compared to conventional imaging methods such as X-ray computed tomography (CT) and magnetic resonance imaging (MRI). 2-[^18^F]fluoro-2-deoxy-D-glucose-([^18^F]FDG) PET, the clinical reference for monitoring response to therapy in oncology, is dependent on the glycolytic metabolic rate of viable tumor cells (Warburg effect) (Kelloff et al. 2005; Kostakoglu and Goldsmith 2003). After effective cancer therapy, a decrease in tumor metabolic activity and subsequent lower [^18^F]FDG uptake is observed. Besides physiological biodistribution, [^18^F]FDG also accumulates in activated macrophages and other inflammatory cells that infiltrate into solid tumors after the start of certain cancer therapies (e.g., immunotherapy). The distinction between FDG uptake due to post-treatment inflammation and tumor cell viability is not always apparent and may complicate response assessment (Ben-Haim and Ell 2009; Subbiah et al. 2017). Therefore, a precise assessment of tumor response can take several months, and more specific tracers are urgently required (Boellaard et al. 2015).

As many cancer treatments work by the induction of apoptosis and/or necrosis, imaging of cell death could represent a useful alternative for [^18^F]FDG for early treatment response evaluation. Several SPECT and PET cell death imaging radiotracers have been developed (Rybczynska et al. 2018). However, their application has been largely limited to preclinical research. Given the crucial role of caspase-3 in initiating the apoptotic pathway, PET imaging of caspase-3 activation has been identified as an attractive approach to predict response to anticancer therapy and has been studied in preliminary clinical trials (Challapalli et al. 2013; Doss et al. 2013; Dubash et al. 2018a). [^18^F]CP18 is a radiolabeled caspase-3 substrate that crosses the cell membrane, and undergoes intracellular trapping upon caspase cleavage. This metabolic trapping represents an important advantage of [^18^F]CP18 because it can result in signal enhancement. The radiotracer was found to accumulate specifically in cultured tumor cells after treatment with 5-FU in good correlation with caspase-3/-7 activity (Su et al. 2013). However, in an in vivo PET imaging study for the early detection of chemotherapy-induced tumor apoptosis, [^18^F]CP18 showed a modest increase in tracer uptake in drug-treated xenograft model of colorectal cancer (Rapic et al. 2017). Nevertheless, [^18^F]CP18 has been evaluated in a clinical trial in healthy human volunteers, in which the tracer showed a fast clearance via the kidneys, and a safe dosimetry profile (Doss et al. 2013). No further patient studies have been reported so far.

Another promising approach to image caspase-3 activity employs the use of selective and high affinity caspase inhibitors, so called activity-based probes (ABPs). One of the most promising small-molecule caspase-3-targeting ABPs, [^18^F]ICMT-11, was extensively evaluated preclinically, where it showed the ability to detect tumor response to different anticancer therapies in several tumor models. The promising preclinical results supported its translation into clinical evaluation of biodistribution and dosimetry in healthy volunteers. Here, despite being considered safe, [^18^F]ICMT-11 has shown a suboptimal pharmacokinetic profile, with high retention of radioactivity in the liver and intestines, which may limit its value for imaging the abdominal region (Challapalli et al. 2013). Furthermore, the tracer was evaluated in a small cohort of breast and lung cancer patients, before and 24 h and 7 days after the first cycle of first-line chemotherapy (Dubash et al. 2018a). Overall, low tumor uptake was observed and no changes from baseline were visible in these patients, which was attributed to a lack of apoptosis induction by the treatment and a heterogeneous response to therapy within tumors. Moreover, most breast cancer patients had necrotic tumors, explaining the low levels of tracer uptake.

These results underline potential limitations of both substrate and activity-based radiotracers for monitoring treatment response. The failure to detect therapy-induced necrosis, contributes to lower signal sensitivity. In addition, given the dynamic nature of caspase-3 activation, monitoring of apoptosis using these tracers is time-dependent. Thus, selecting the appropriate time window for evaluating tumor response is important (Nguyen et al. 2013). Another target to image the apoptotic process involves the redistribution of phospholipids in the cell membrane during the early stages of cell death. The aminophospholipids phosphatidylethanolamine (PE) and phosphatidylserine (PS) primarily reside in the inner leaflet of the membrane in viable mammalian cells but are transported during the process of apoptosis to the outer leaflet (Suzuki and Nagata 2014). Because the rupture of the plasma membrane during necrosis also results in accessible PS and PE, both phospholipids represent high abundance targets for imaging both apoptosis and necrosis.

The most studied PS-binding protein is annexin V. Several radiolabeled annexin V analogs have been developed and evaluated clinically, most notably [^99m^Tc]HYNIC-annexin V. Overall, studies with this tracer showed that annexin V has the capability for binding apoptotic tumor cells and the potential for evaluating tumor response. However, the protein-nature of annexin V-based radiotracers resulted in suboptimal biodistribution profiles with slow blood clearance and high background activity in the abdominal region and limited tumor penetration (Kemerink et al. 2001). Several studies using radiolabeled annexin V have highlighted the importance of defining an optimal imaging window, where the radiotracer uptake in tumors appears to be heavily dependent on the timing of imaging after the start of a given therapy (Belhocine et al. 2004; Blankenberg 2008, 2002).

An attractive alternative to annexin V-based cell death imaging is presented by the lantibiotic probe duramycin, which exhibits a high affinity and specificity for PE (Zhao 2011; Iwamoto et al. 2007). The low molecular weight of duramycin (approximately 2 kDa) enables rapid clearance from the bloodstream, minimal non-target uptake, and efficient penetration into target tissues (Mosayebnia et al. 2020; Hosseinimehr 2020). The higher availability of PE in the plasma membrane, represents another advantage of duramycin over annexin V (Vance 2008). Duramycin has been labeled with ^99m^Tc using the bifunctional chelating agent hydrazinonicotinamide (HYNIC) using a kit formulation, enabling its use for SPECT imaging. A μSPECT imaging study showed favorable dosimetry for [^99m^Tc]Tc-Duramycin as well as rapid renal clearance and low blood pool and hepatic background (Zhao et al. 2008). Because of these good imaging characteristics, the usefulness of [^99m^Tc]Tc-Duramycin to assess early treatment response was demonstrated in human colorectal cancer xenografts using different therapeutic strategies, including cytotoxic drugs, radiotherapy and targeted therapy, all directed at promoting tumor cell death (Johnson et al. 2013; Elvas et al. 2017, 2015; Li et al. 2018; Liu et al. 2020). Of particular significance, [^99m^Tc]Tc-Duramycin was able to differentiate treatment-responding from non-responding tumors in colorectal cancer xenografts in only 24 h after initiating targeted therapy, while [^18^F]FDG uptake was not (Elvas et al. 2017). All these findings collectively indicate that [^99m^Tc]Tc-Duramycin warrants further investigation for early treatment response evaluation in cancer patients.

The goal of the current study was to evaluate the safety, pharmacokinetics, and internal dosimetry of [^99m^Tc]Tc-Duramycin in healthy human volunteers.

## Methods

### Subjects and safety precautions

Six healthy volunteers aged 18 years (y) or older were included after providing written informed consent in accordance with the Declaration of Helsinki guidelines. Exclusion criteria encompassed chronic diseases, pregnancy, lactation, metal implants, body weight over 100 kg, severe claustrophobia, and abnormal kidney or liver function. Ethical approval was obtained from the local ethics committee (Trial Number: NCT05177640).

Prior to the study, volunteers underwent a medical history review, collection of blood samples for clinical laboratory chemistry (renal and liver function, hematology and blood coagulation parameters), a 12-lead electrocardiogram, and a physical examination. Volunteers were hospitalized for 24 h to perform all scans and monitor vital signs at different time points. Pregnancy was ruled out in female volunteers through human chorionic gonadotropin hormone measurement prior to tracer injection. Clinical laboratory chemistry was repeated 6 h after tracer injection, and an electrocardiogram was conducted prior to discharge. Three days after the scans, a medical visit was scheduled for a final physical examination and collection of a blood sample for chemistry. Finally, volunteers were contacted by phone after 1 month to assess any delayed reactions.

### Radiopharmaceutical preparation

[^99m^Tc]Tc-Duramycin was manufactured using a method developed by our group (Palmieri et al. 2018). HYNIC-duramycin was provided by Primera Analytical Solutions Corp. (USA). A good manufacturing practice-(GMP) grade radiolabeling kit containing HYNIC-duramycin, tricine and Tris(3-sulfophenyl)phosphine trisodium salt (TPPTS), and SnCl_2_·2H_2_O, was prepared at University of Iowa Pharmaceuticals (USA) in compliance with GMP, according to a known procedure (Zhao and Li 2012). Labeling with ^99m^Tc was performed by addition of [^99m^Tc]NaTcO_4_ (1.85 GBq) to the radiolabeling kit, immediately followed by heating at 80 °C for 20 min. After cooling to 25 °C, the reaction mixture was purified using solid-phase extraction (SPE), as previously described (Palmieri et al. 2018). Briefly, the reaction mixture was trapped in a SPE cartridge (Waters Oasis HLB Plus Short, 225 mg, 60 μm, USA), followed by a wash step with 5 mL of water for injection and elution with 2 mL 96% ethanol. Finally, [^99m^Tc]Tc-Duramycin was formulated as a 46.25 MBq/mL 0.9% NaCl/Ethanol solution by addition of 18 mL sterile 0.9% NaCl to the previous solution. Before injection, the preparation was passed through a sterile 0.22-μm filter. The radiochemical purity (RCP) was determined by instant thin-layer chromatography and analytical HPLC, as described before (Elvas et al. 2015). In addition, endotoxin and sterility analysis were performed according to the methods described in the European Pharmacopoeia. Full release specifications and validation batch analysis are shown in Additional file 1: Table S1.

### Data acquisition

Images were acquired on a dual-detector SPECT/16 slice CT scanner (Discovery NM/CT 670, GE) with low-energy, high-resolution, parallel-hole collimators, that was cross-calibrated with the radionuclide dose calibrator and the automated gamma-counter.

Before injection of [^99m^Tc]Tc-Duramycin, a whole body (WB) transmission scan was performed using a ^57^Co sheet source to obtain a photon attenuation map of each volunteer. Dynamic planar images of the mid-thorax and upper abdominal region were acquired immediately upon radiotracer injection (30 frames of 1 min) to visualize initial activity distribution.

WB planar images were collected at 1 h, 2 h, 3 h, 6 h, and 23 h post-injection (PI). During each WB acquisition, a syringe with a known activity of ^99m^Tc was placed between the volunteers' feet for cross-calibration between the radionuclide dose calibrator and gamma-camera. Attenuation correction was done per organ, self-attenuation and scattering estimates were not included. The WB attenuation correction was done by matching with the total injected activity at the first WB image as the volunteers were requested not to void their bladders before the first scan.

After each WB planar scan, a SPECT scan consisting of one bed position (60 projections, 25 s per projection), over the abdomen was performed to collect data on the activity distribution in the liver, kidneys and spleen. The SPECT scans were corrected for attenuation using one low-dose CT scan (120 kV, Auto mA, Noise Index 21) performed immediately after the first SPECT scan.

Blood samples were collected before the first injection and 5 min, 10 min, 15 min, 30 min, 1 h, 1.5 h, 3 h, 6 h and 23 h PI and residual radioactivity was measured in a standardized manner using a gamma-counter. Plasma samples for metabolite analysis and protein binding were collected at 5, 30, 60, 180 and 360 min after tracer injection and analyzed as previously described (Elvas et al. 2015). Values for plasma binding were expressed as % free radiotracer.

Urine and feces were collected during the 23 h PI. After weighing and homogenization, the samples were measured in a gamma counter.

### Data analysis

The regions of interest (ROIs) were delineated manually on the 3 h WB planar image and transferred to all other planar images including the transmission scans to determine the uptake of the radiotracer. The WB ROI was considered as the entire image minus the standard between the volunteers’ feet. The following source regions were included: WB, liver, kidneys, lungs, spleen, heart, brain, and bladder. For the SPECT reconstruction, attenuation correction, scatter correction and collimator modeling were applied in the iterative image reconstruction (4 iterations, 16 subsets). A semi-automated approach using an AI contouring tool (MIM Software) was used to delineate the ROIs, which were manually corrected where needed.

At each time point, the percentage injected dose (%ID) of each source organ was calculated according to the formula:

$$\% ID_{source\, organ} \left( t \right) = \frac{{A_{source\, organ} \left( t \right)}}{{A_{injected\, dose} }} \times 100.$$

The time-activity curves for the whole body, selected organs and blood were generated and fitted in MATLAB (MathWorks Inc.) using a single exponential function:

$$A = ae^{ - bt} ,$$

where A is the measured activity at time point t. The integral of this time-activity curve (= a/b) normalized for the injected activity was used as input for the dose calculation using IDAC-Dose 2.1 (Andersson et al. 2017). This allowed for a dose calculation using ICRP 103 (Valentin 2007).

A two-compartment model was used for kinetic modeling of the blood data using a bi-exponential function: $A = ae^{ - bt} + ce^{ - dt}$.

## Results

### Subjects and safety

The mean age of the 6 volunteers was 30 years (range, 24–46 years), and their mean bodyweight was 80 kg (range, 74.6–91.3 kg). The mean injected [^99m^Tc]Tc-Duramycin activity in all volunteers was 473 ± 36 MBq (range, 436–533 MBq). The overall radiochemical purity was 97.38 ± 1.22% (n = 6), with a minor polar compound remaining in the formulation after SPE purification. [^99m^Tc]Tc-Duramycin was safe and well tolerated in all subjects. No tracer-related serious adverse events (AEs) were observed. Two volunteers had minor AEs (grade 1). One subject had marginally elevated D-dimers 6 h after tracer injection, without clinical signs of thrombosis and with normalization at 23 h. Another volunteer had an injection site reaction that was present up to 3 days after the removal of the catheter. No significant changes in vital signs, other clinical laboratory blood tests, or electrocardiograms were observed.

### Biodistribution

**Fig. 1:**
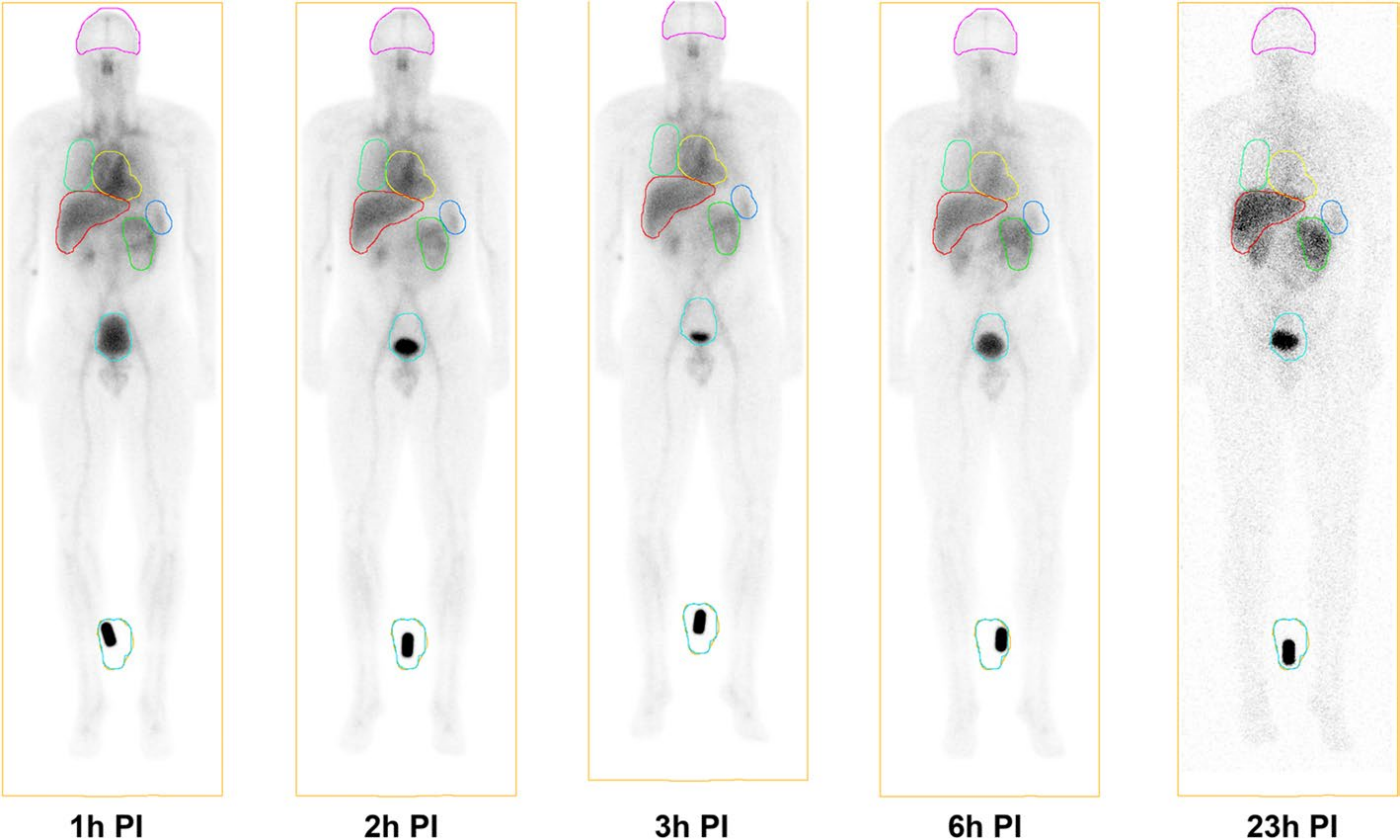
Anterior planar decay-corrected whole-body images of a volunteer from 1 to 23 h after [^99m^Tc]Tc-Duramycin injection. All other subjects had a similar distribution

**Fig. 2:**
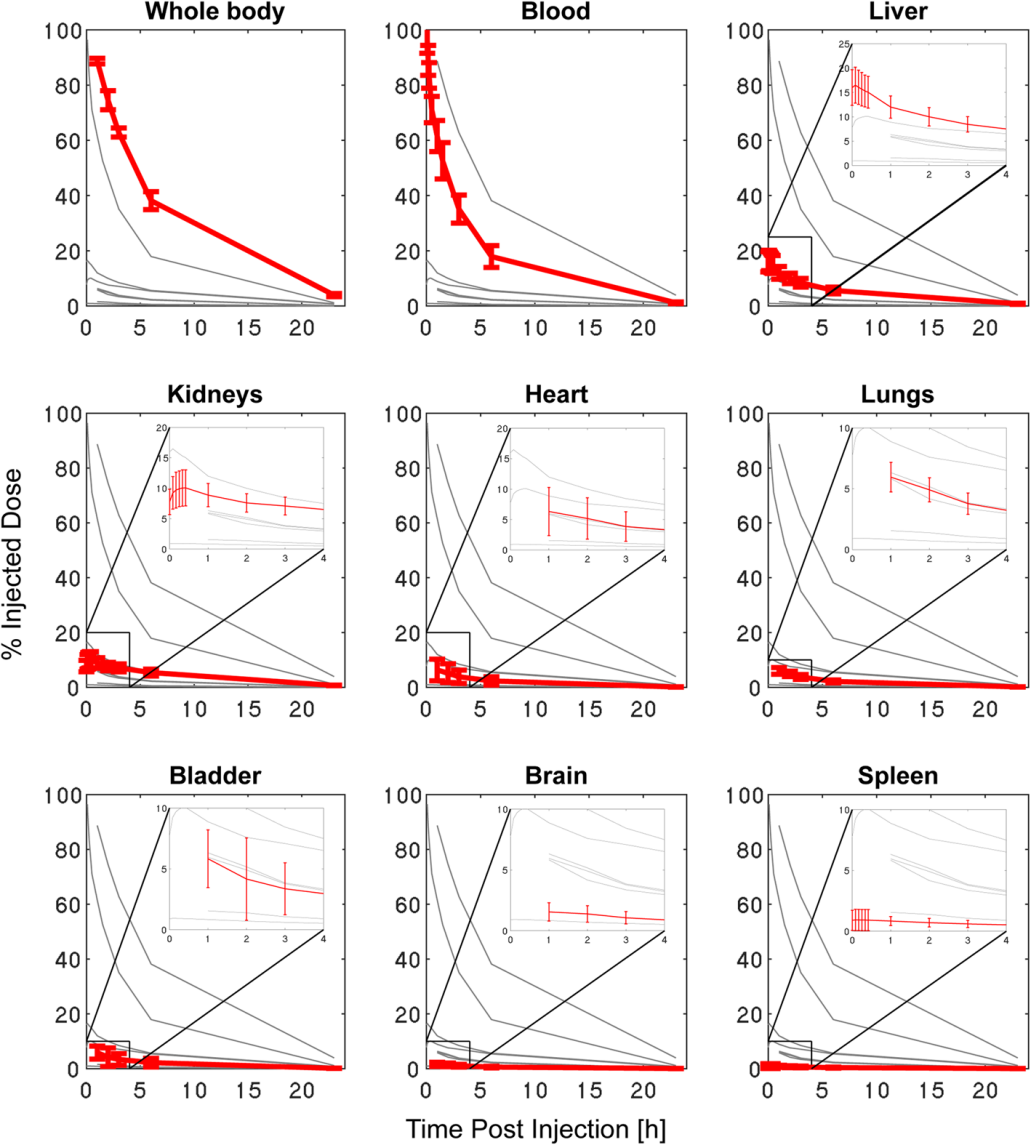
Mean percentage injected dose (%ID) for all regions-of-interest from the planar scans of the 6 volunteers, as a function of time after injection. Error bars indicate one standard deviation (SD)

After administration of [^99m^Tc]Tc-Duramycin, radioactivity was detected in the vascular compartment and rapidly distributed to the liver and kidneys (Fig. 1). Uptake in the liver was faster compared to the kidneys, with peak activity at 5 min PI (16.49 ± 3.69%ID), versus 25 min PI in the kidneys (10.06 ± 2.96%ID) (Fig. 2). Additionally, the clearance was faster in the liver with an effective half-life of 3.30 h for the liver versus 6.91 h for the kidneys. The clearance rate for the kidneys was 0.14%/min. About 56% of the injected activity was eliminated within the first 24 h through the kidneys, and no hepatobiliary excretion was observed (0.2%ID in the feces after 24 h). There was no significant uptake in any brain structure. The clearance of activity from the blood pool was slow (effective half-life of 2.03 h), as shown by the visualization of several major arteries up to 6 h PI (Fig. 1). The results of the kinetic modeling showed a fast component with a fraction of 47 ± 11% eliminated with a half-life of 48 ± 9 min and a slow component with a fraction of 51 ± 9% eliminated with a half-life of 12 ± 1 h. As a result, the WB retention was high with an effective half-life of 4.15 h and corresponding biological half-life of 14.67 h.

**Fig. 3:**
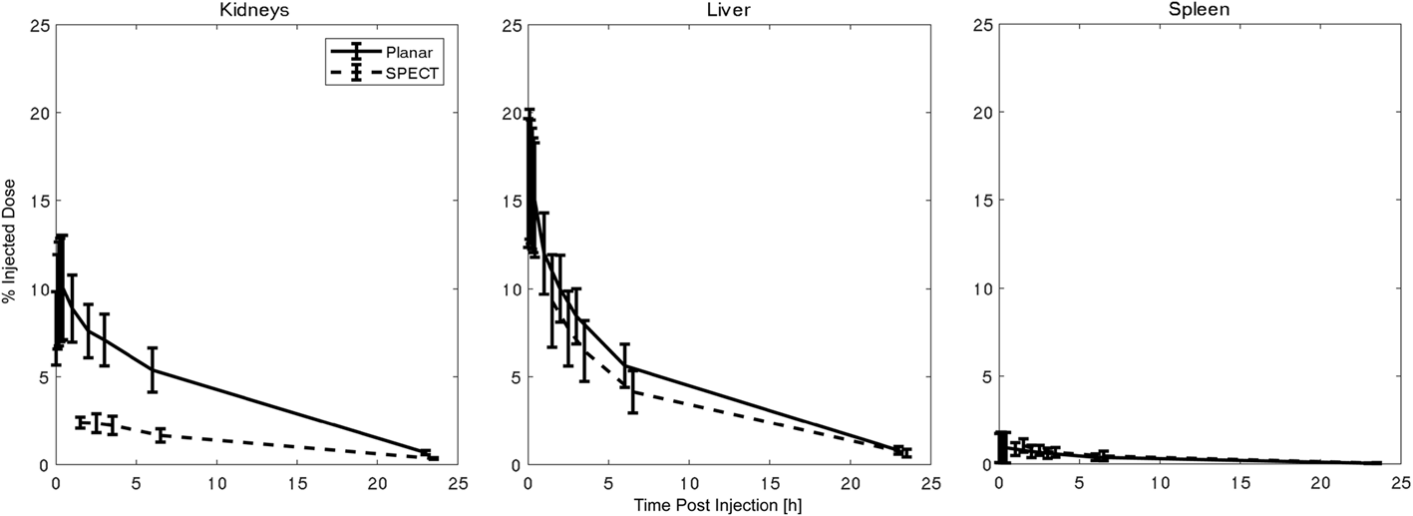
Side-by-side comparison of the mean percentage injected dose for the 6 volunteers obtained by SPECT and planar image analysis for kidneys, liver, and spleen. Error bars indicate one SD

Quantification of radioactivity uptake values in organs was different between planar and SPECT images (Fig. 3), with lower activity measured in the kidneys on SPECT (peak 2.4 ± 0.37%ID) vs. the planar images (peak 9.29 ± 2.62%ID). There were no differences in the biodistribution profiles between male and female volunteers. The mean residence times (RT) averaged over male and female subjects are summarized in Table 1, with the liver showing the highest RT. The individual half-lives and RT for each volunteer are described in Additional file 1: Tables S2 and S3.

**Table 1:** Mean residence times (h) for [^99m^Tc]Tc-Duramycin calculated from the planar images for the different organs averaged over male and female subjects

Target organs	Residence times (h)
Heart	0.37 ± 0.24
Kidneys	0.34 ± 0.08
Liver	0.81 ± 0.20
Urinary bladder	0.35 ± 0.25
Whole body	6.32 ± 0.49

Values are expressed as mean ± SD

### Dosimetry

**Fig. 4:**
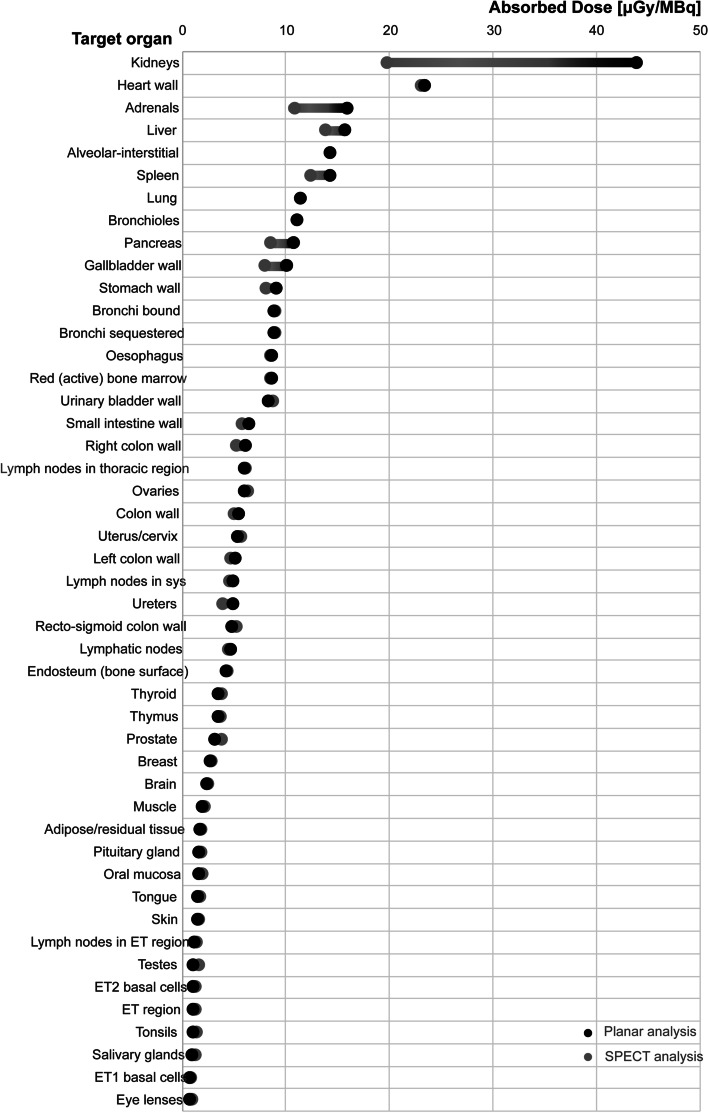
Comparison of the organ absorbed doses between values obtained from SPECT and planar images

Table 2 summarizes the mean organ absorbed dose estimates for [^99m^Tc]Tc-Duramycin. The kidneys (43.8 ± 4.07), heart wall (23.5 ± 7.75), liver (15.7 ± 2.33), and spleen (14.3 ± 1.67) showed the highest absorbed dose (µGy/MBq) among all organs based on WB planar images. The mean effective dose averaged over male and female subjects was estimated to be 7.61 ± 0.77 µSv/MBq. When the dose estimation was based on values from the SPECT images, the absorbed organ dose was overall lower, and the effective dose was 7.10 ± 0.67 µGy/MBq (Fig. 4; Table 2).

**Table 2:** Mean organ absorbed dose calculations using the ICRP 103 model as implemented in IDAC-Dose 2.1

Organs	SPECT analysis	Planar analysis
Adipose/residual tissue	1.76 ± 0.07	1.69 ± 0.10
Adrenals	10.81 ± 1.30	15.88 ± 1.64
Alveolar-interstitial	14.20 ± 1.76	14.25 ± 1.72
Brain	2.48 ± 0.85	2.37 ± 0.87
Breast	2.74 ± 0.24	2.64 ± 0.24
Bronchi bound	8.96 ± 1.24	8.88 ± 1.22
Bronchi sequestered	8.95 ± 1.24	8.88 ± 1.22
Bronchioles	11.04 ± 1.39	11.07 ± 1.35
Colon wall	5.03 ± 0.24	5.46 ± 0.37
Endosteum (bone surface)	4.36 ± 0.78	4.18 ± 0.86
ET region	1.30 ± 0.17	1.03 ± 0.22
ET1 basal cells	0.85 ± 0.12	0.67 ± 0.16
ET2 basal cells	1.30 ± 0.17	1.03 ± 0.22
Eye lenses	0.87 ± 0.13	0.65 ± 0.17
Gallbladder wall	7.98 ± 1.42	10.11 ± 0.97
**Heart wall**	**23.10 ± 7.64**	**23.35 ± 7.75**
**Kidneys**	**19.72 ± 3.42**	**43.82 ± 4.07**
Left colon wall	4.71 ± 0.32	5.13 ± 0.63
**Liver**	**13.83 ± 3.18**	**15.70 ± 2.33**
Lung	11.42 ± 1.38	11.38 ± 1.32
Lymphatic nodes	4.46 ± 0.27	4.66 ± 0.28
Lymph nodes in ET region	1.37 ± 0.31	1.15 ± 0.27
Lymph nodes in sys	4.59 ± 0.29	4.87 ± 0.31
Lymph nodes in thoracic region	6.13 ± 0.90	6.02 ± 0.90
Muscle	2.10 ± 0.18	1.94 ± 0.24
Oesophagus	8.56 ± 1.05	8.60 ± 1.08
Oral mucosa	1.86 ± 0.20	1.53 ± 0.26
Ovaries	6.34 ± 3.23	6.02 ± 3.06
Pancreas	8.54 ± 0.91	10.68 ± 0.73
Pituitary gland	1.81 ± 0.44	1.55 ± 0.48
Prostate	3.81 ± 2.09	3.16 ± 1.81
Recto-sigmoid colon wall	5.20 ± 1.04	4.81 ± 1.08
**Red (active) bone marrow**	**8.49 ± 1.82**	**8.59 ± 1.82**
Right colon wall	5.26 ± 0.40	6.12 ± 0.62
Salivary glands	1.21 ± 0.16	0.90 ± 0.22
Skin	1.62 ± 0.19	1.46 ± 0.23
Small intestine wall	5.80 ± 0.72	6.45 ± 0.69
Spleen	12.37 ± 2.31	14.25 ± 1.67
Stomach wall	8.03 ± 0.88	9.01 ± 0.76
Testes	1.61 ± 0.81	1.08 ± 0.54
Thymus	3.71 ± 0.44	3.44 ± 0.40
Thyroid	3.76 ± 0.35	3.45 ± 0.41
Tongue	1.74 ± 0.20	1.50 ± 0.23
Tonsils	1.38 ± 0.20	1.03 ± 0.23
Ureters	3.84 ± 0.47	4.87 ± 0.41
Urinary bladder wall	8.72 ± 4.25	8.31 ± 4.27
Uterus/cervix	5.67 ± 3.25	5.36 ± 3.07
**Effective dose 103 [mSv/MBq]**	**7.10 ± 0.67**	**7.61 ± 0.75**

For the individual organ doses (expressed in µGy/MBq), the highest dose of either the male or female dose is included in the table. Data are mean ± SD; n = 6

Highest mean organ absorbed doses and effective dose are presented in bold

### Metabolite and protein binding analysis

**Fig. 5:**
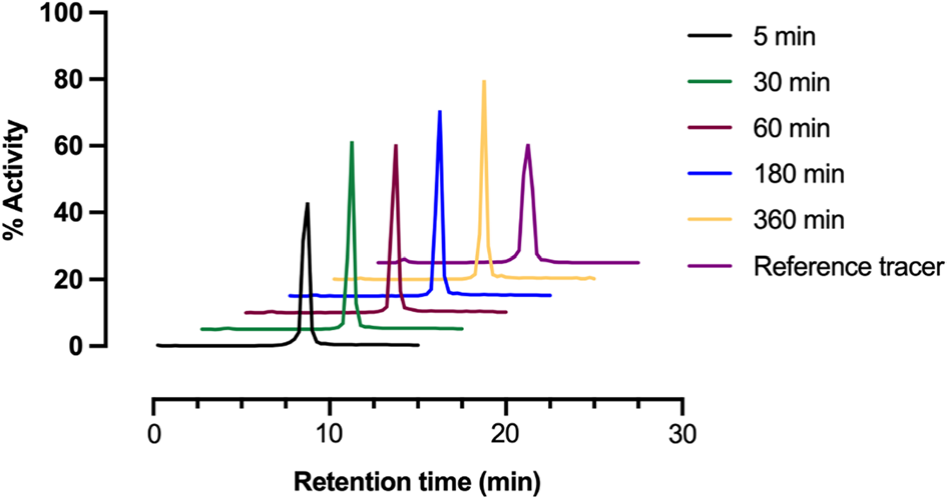
Stability of [^99m^Tc]Tc-Duramycin after injection in human volunteers. Representative radio-HPLC chromatograms of a human plasma sample after [^99m^Tc]Tc-Duramycin injection

The analysis of the plasma samples showed that [^99m^Tc]Tc-Duramycin remained mostly intact up to 360 min after injection (93 ± 3%). The radio-HPLC analysis detected the intact parent radiotracer and one minor polar metabolite (Fig. 5). This radiolabeled species (∼ 2%) was also present in the reference tracer radio-HPLC chromatogram. Therefore it was not related to in vivo metabolization of [^99m^Tc]Tc-Duramycin. This pattern is consistent with what was previously observed in preclinical studies (Elvas et al. 2015).

Binding of [^99m^Tc]Tc-Duramycin to plasma proteins was analyzed after injection. A high association to plasma proteins was found (Additional file 1: Table S4). The mean percentage of free radiotracer in healthy subjects decreased from 3.4 ± 1.5% to 2.2 ± 0.8% from 5 to 1440 min after injection.

## Discussion

We have shown in this first-in-human study that [^99m^Tc]Tc-Duramycin is safe and well tolerated in healthy human volunteers. The cell death imaging tracer showed a long retention in the blood with predominantly renal elimination of activity at a clearance rate of 0.14%/min. In addition, high uptake was also found in the liver and spleen. Optimal target-to-background ratios are expected 5 h PI. However, the prolonged retention in the liver and spleen even after 23 h PI, could hamper tumor response assessment in these organs.

We determined the mean effective dose of [^99m^Tc]Tc-Duramycin as 7.6 µSv/MBq, corresponding to a total effective dose of 3.60 mSv for a patient dose of 473 MBq. This value compares favorably to those for other cell death imaging tracers such as [^18^F]ICMT-11 (12.5 mSv), [^18^F]CP18 (2.8 mSv), [^99m^Tc]HYNIC-annexin V (5.5 mSv) or [^18^F]ML-10 (7.7 mSv), and is comparable with the effective dose of [^18^F]FDG (9.0 mSv) (Challapalli et al. 2013; Doss et al. 2013; Hoglund et al. 2011; Kemerink et al. 2003; Quinn et al. 2016). Moreover, this dosimetry study showed that [^99m^Tc]Tc-Duramycin meets the criteria for radiation exposure specified by the European guidelines and US Food and Drug Administration.

Consistent with the predominant renal excretion of [^99m^Tc]Tc-duramycin, the kidney was the dose-limiting organ (23 µSv/MBq). Therefore, good hydration and frequent bladder voiding are recommended in future patient studies. Another organ receiving a relatively high dose was the heart wall (20 and 44 µGy/MBq for SPECT and planar imaging, respectively), reflecting slower blood clearance and high radiotracer association with plasma proteins, such as lipoproteins that are known to contain PE (Zhao et al. 2008). Protein-bound radiotracer is less likely to partition across capillary membranes into tissues when compared with free radiotracer in plasma. However, the equilibrium between bound and free radiotracer in the plasma is usually rapid; therefore, most of the protein-bound radiotracer is expected to become available to the tissue (Marc 2009).

The biodistribution of [^99m^Tc]Tc-Duramycin in human subjects was consistent with previous results in mice showing slow blood clearance, and high liver and spleen accumulation, which can potentially limit tumor response assessment in these organs (Elvas et al. 2015; Palmieri et al. 2018). The latter was suggested to be attributable to the increased retention of the radiotracer in the reticuloendothelial cells in the liver and spleen. We have found that HPLC purification of kit-prepared [^99m^Tc]Tc-Duramycin resulted in better imaging properties with faster clearance from the blood and lower uptake in the liver. In addition, in animal studies, we have demonstrated that this need for purification of kit-prepared [^99m^Tc]Tc-Duramycin is a species-related phenomenon (Palmieri et al. 2018). For human use, producing a radiotracer under GMP compliance is essential. Therefore, in this study we have chosen a production method for [^99m^Tc]Tc-Duramycin using SPE cartridges, which has an easier technical implementation compared to the HPLC method. However, using the SPE purified [^99m^Tc]Tc-Duramycin in humans, we could not obtain a reduced liver and spleen accumulation like previously demonstrated in mice with the HPLC purified tracer (Marc 2009). Further research is needed, and we will consider the possibility of utilizing HPLC purification in future human studies, if necessary.

Response to cancer therapy is associated with the induction of various cell death molecular mechanisms. Since [^99m^Tc]Tc-Duramycin can bind both apoptotic and necrotic tumor cells, it is likely that PE accessible in necrotic cells might contribute to an increased signal observed in the tumors. In addition, the higher availability of PE binding sites represents a clear advantage of duramycin over PS binding counterparts, such as annexin V, as PS is a representative minor constituent of the plasma membrane (Vance 2008). In a comparison study with [^99m^Tc]HYNIC-annexin V, [^99m^Tc]Tc-Duramycin showed higher affinity to apoptotic cells and improved uptake in apoptotic atherosclerotic lesions (Hu et al. 2018).

Imaging agents that target activated caspase-3, an executioner caspase in the apoptosis pathway, may suffer from additional limitations for detecting of cell death in vivo. Since caspase-3 activation is a fast and transient process, following treatment, it is necessary to select appropriate timing for imaging carefully (Dubash et al. 2018b). Although the exposure of PE in the cell surface is a more stable marker during the whole apoptosis process, the choice of the temporal imaging window following treatment is also critical for [^99m^Tc]Tc-Duramycin. Therefore, similarly to other cell death imaging tracers, we may find it beneficial to use multiple imaging time points after radiotracer injection, allowing greater tracer accumulation in apoptotic cells and the detection of peak apoptotic activity, therefore improving our assessment of therapeutic efficacy.

The next step is to assess the ability of [^99m^Tc]Tc-Duramycin to assess tumor response in cancer patients. Currently, a phase II study (EudraCT 2023-000064-64) is ongoing in which newly diagnosed metastatic cancer patients will undergo a [^99m^Tc]Tc-Duramycin scan before and 2 and 7 days after the start of first-line systematic therapy (chemotherapy, targeted therapy, immunotherapy, or combination of both). Changes in [^99m^Tc]Tc-Duramycin uptake in different organ metastases will be correlated with [^18^F]FDG response after 7 days and the best overall RECIST response after 6 months. Given the high physiological background uptake in liver, the ability to detect response in liver metastases could be hampered. Therefore, an interim analysis after 10 patients with liver metastases is scheduled. If less than 30% of the responding liver lesions show an increased [^99m^Tc]Tc-Duramycin uptake, a switch from SPE to HPLC tracer purification will be made in the remaining patients in an attempt to reduce the non-specific liver uptake. Results of this clinical study will provide crucial insights into the radiopharmaceutical's clinical performance and its potential to improve patient outcomes. Ultimately, these evaluations will determine whether the radiopharmaceutical can be successfully translated into routine clinical practice as a valuable tool for cancer treatment planning and monitoring.

## Conclusion

[^99m^Tc]Tc-Duramycin was well tolerated in all subjects, and did not cause significant side effects. Despite higher radiation dose, when compared with the estimates obtained from animal studies, [^99m^Tc]Tc-Duramycin showed a favorable radiation dosimetry profile, comparable or lower than other cell death imaging agents. These results support the further validation of [^99m^Tc]Tc-Duramycin for clinical imaging of treatment-induced cell death in cancer patients.

### Supplementary Information

**Additional file 1**. contains supplementary data with the release specifications of [^99m^Tc]Tc-Duramycin, the individual effective half-lives and residence times for all healthy volunteers and the plasma protein binding analysis.

## Data Availability

All data generated or analyzed during this study are included in this published article and its supplementary information file.

## References

[CR1] Andersson M, Johansson L, Eckerman K, Mattsson S (2017). IDAC-Dose 2.1, an internal dosimetry program for diagnostic nuclear medicine based on the ICRP adult reference voxel phantoms. EJNMMI Res.

[CR2] Belhocine T, Steinmetz N, Li C, Green A, Blankenberg FG (2004). The imaging of apoptosis with the radiolabeled annexin V: optimal timing for clinical feasibility. Technol Cancer Res Treat.

[CR3] Ben-Haim S, Ell P (2009). 18F-FDG PET and PET/CT in the evaluation of cancer treatment response. J Nucl Med off Publ Soc Nucl Med.

[CR4] Blankenberg F (2002). To scan or not to scan, it is a question of timing: technetium-99m-annexin V radionuclide imaging assessment of treatment efficacy after one course of chemotherapy. Clin Cancer Res.

[CR5] Blankenberg FG (2008). In vivo detection of apoptosis. J Nucl Med off Publ Soc Nucl Med.

[CR6] Boellaard R, Delgado-Bolton R, Oyen WJ, Giammarile F, Tatsch K, Eschner W (2015). FDG PET/CT: EANM procedure guidelines for tumour imaging: version 2.0. Eur J Nucl Med Mol Imaging.

[CR7] Challapalli A, Kenny LM, Hallett WA, Kozlowski K, Tomasi G, Gudi M (2013). 18F-ICMT-11, a caspase-3-specific PET tracer for apoptosis: biodistribution and radiation dosimetry. J Nucl Med off Publ Soc Nucl Med.

[CR8] Doss M, Kolb HC, Walsh JC, Mocharla V, Fan H, Chaudhary A (2013). Biodistribution and radiation dosimetry of 18F-CP-18, a potential apoptosis imaging agent, as determined from PET/CT scans in healthy volunteers. J Nucl Med off Publ Soc Nucl Med.

[CR9] Dubash SR, Merchant S, Heinzmann K, Mauri F, Lavdas I, Inglese M (2018). Clinical translation of [(18)F]ICMT-11 for measuring chemotherapy-induced caspase 3/7 activation in breast and lung cancer. Eur J Nucl Med Mol Imaging.

[CR10] Dubash SR, Merchant S, Heinzmann K, Mauri F, Lavdas I, Inglese M (2018). Clinical translation of [18F]ICMT-11 for measuring chemotherapy-induced caspase 3/7 activation in breast and lung cancer. Eur J Nucl Med Mol Imaging.

[CR11] Elvas F, Vangestel C, Rapic S, Verhaeghe J, Gray B, Pak K (2015). Characterization of [(99m)Tc]Duramycin as a SPECT imaging agent for early assessment of tumor apoptosis. Mol Imaging Biol MIB off Publ Acad Mol Imaging.

[CR12] Elvas F, Boddaert J, Vangestel C, Pak K, Gray B, Kumar-Singh S (2017). (99m)Tc-Duramycin SPECT imaging of early tumor response to targeted therapy: a comparison with (18)F-FDG PET. J Nucl Med off Publ Soc Nucl Med.

[CR13] Hoglund J, Shirvan A, Antoni G, Gustavsson SA, Langstrom B, Ringheim A (2011). 18F-ML-10, a PET tracer for apoptosis: first human study. J Nucl Med off Publ Soc Nucl Med.

[CR14] Hosseinimehr SJ (2020). Radiolabeled peptides in imaging and therapy: basic and clinical perspectives. Curr Med Chem.

[CR15] Hu Y, Liu G, Zhang H, Li Y, Gray BD, Pak KY (2018). A comparison of [99mTc]duramycin and [99mTc]annexin V in SPECT/CT imaging atherosclerotic plaques. Mol Imaging Biol.

[CR16] Iwamoto K, Hayakawa T, Murate M, Makino A, Ito K, Fujisawa T (2007). Curvature-dependent recognition of ethanolamine phospholipids by duramycin and cinnamycin. Biophys J.

[CR17] Johnson SE, Li Z, Liu Y, Moulder JE, Zhao M (2013). Whole-body imaging of high-dose ionizing irradiation-induced tissue injuries using 99mTc-duramycin. J Nucl Med off Publ Soc Nucl Med.

[CR18] Kelloff GJ, Hoffman JM, Johnson B, Scher HI, Siegel BA, Cheng EY (2005). Progress and promise of FDG-PET imaging for cancer patient management and oncologic drug development. Clin Cancer Res.

[CR19] Kemerink GJ, Boersma HH, Thimister PW, Hofstra L, Liem I, Pakbiers MT (2001). Biodistribution and dosimetry of (99m)Tc-BTAP-annexin-V in humans. Eur J Nucl Med.

[CR20] Kemerink GJ, Liu X, Kieffer D, Ceyssens S, Mortelmans L, Verbruggen AM (2003). Safety, biodistribution, and dosimetry of 99mTc-HYNIC-annexin V, a novel human recombinant annexin V for human application. J Nucl Med off Publ Soc Nucl Med.

[CR21] Kostakoglu L, Goldsmith SJ (2003). 18F-FDG PET evaluation of the response to therapy for lymphoma and for breast, lung, and colorectal carcinoma. J Nucl Med off Publ Soc Nucl Med.

[CR22] Li Y, Liu C, Xu X, Lu X, Luo J, Gray B (2018). [(99m)Tc]Tc-duramycin, a potential molecular probe for early prediction of tumor response after chemotherapy. Nucl Med Biol.

[CR23] Liu C, Li Y, Qin X, Yang Z, Luo J, Zhang J (2020). Early prediction of tumor response after radiotherapy in combination with cetuximab in nasopharyngeal carcinoma using (99m) Tc-duramycin imaging. Biomed Pharmacother.

[CR24] Marc SB (2009). The importance of kinetic enhancement. J Nucl Med.

[CR25] Mosayebnia M, Hajiramezanali M, Shahhosseini S (2020). Radiolabeled peptides for molecular imaging of apoptosis. Curr Med Chem.

[CR26] Nguyen QD, Lavdas I, Gubbins J, Smith G, Fortt R, Carroll LS (2013). Temporal and spatial evolution of therapy-induced tumor apoptosis detected by caspase-3-selective molecular imaging. Clin Cancer Res off J Am Assoc Cancer Res.

[CR27] Palmieri L, Elvas F, Vangestel C, Pak K, Gray B, Stroobants S (2018). [99mTc]duramycin for cell death imaging: impact of kit formulation, purification and species difference. Nucl Med Biol.

[CR28] Quinn B, Dauer Z, Pandit-Taskar N, Schoder H, Dauer LT (2016). Radiation dosimetry of 18F-FDG PET/CT: incorporating exam-specific parameters in dose estimates. BMC Med Imaging.

[CR29] Rapic S, Vangestel C, Elvas F, Verhaeghe J, den Wyngaert TV, Wyffels L (2017). Evaluation of [(18)F]CP18 as a substrate-based apoptosis imaging agent for the assessment of early treatment response in oncology. Mol Imaging Biol MIB off Publ Acad Mole Imaging.

[CR30] Rybczynska AA, Boersma HH, de Jong S, Gietema JA, Noordzij W, Dierckx RAJO (2018). Avenues to molecular imaging of dying cells: focus on cancer. Med Res Rev.

[CR31] Su H, Chen G, Gangadharmath U, Gomez LF, Liang Q, Mu F (2013). Evaluation of [(18)F]-CP18 as a PET imaging tracer for apoptosis. Mol Imaging Biol MIB off Publ Acad Mol Imaging.

[CR32] Subbiah V, Chuang HH, Gambhire D, Kairemo K (2017). Defining clinical response criteria and early response criteria for precision oncology: current state-of-the-art and future perspectives. Diagnostics (basel).

[CR33] Suzuki J, Nagata S (2014). Phospholipid scrambling on the plasma membrane. Methods Enzymol.

[CR34] The 2007 recommendations of the international commission on radiological protection. ICRP publication 103. Ann ICRP. 2007;37(2–4):1–332. 10.1016/j.icrp.2007.10.003 18082557

[CR35] Vance JE (2008). Phosphatidylserine and phosphatidylethanolamine in mammalian cells: two metabolically related aminophospholipids. J Lipid Res.

[CR36] Zhao M (2011). Lantibiotics as probes for phosphatidylethanolamine. Amino Acids.

[CR37] Zhao M, Li Z (2012). A single-step kit formulation for the (99m)Tc-labeling of HYNIC-Duramycin. Nucl Med Biol.

[CR38] Zhao M, Li Z, Bugenhagen S (2008). 99mTc-labeled duramycin as a novel phosphatidylethanolamine-binding molecular probe. J Nucl Med off Publ Soc Nucl Med.

