# Supplementary material for: First-in-human study of a novel cell death tracer [99mTc]Tc-Duramycin: safety, biodistribution and radiation dosimetry in healthy volunteers
Source: EJNMMI Radiopharm Chem. 2023 Aug 30;8:20. doi: 10.1186/s41181-023-00207-1 (PMC10468453; doi:10.1186/s41181-023-00207-1)
Supplement: Supplementary file 1 — Additional file 1. contains supplementary data with the release specifications of [99mTc]Tc-Duramycin, the individual effective half-lives and residence times for all healthy volunteers and the plasma protein binding analysis. [file 41181_2023_207_MOESM1_ESM.docx]

**Supplementary information**

**First-in-human study of a novel cell death tracer [^99m^Tc]Tc-Duramycin: safety, biodistribution and radiation dosimetry in healthy volunteers**

Taco M. Cappenberg*^1^, Stijn De Schepper*^1^, Christel Vangestel^1,2^, Stef De Lombaerde^1,2^, Leonie wyffels^1,2^, Tim Van den Wyngaert^1,2^, Jeffrey Mattis^3^, Brian Gray^3^, Koon Pak^3^, Sigrid Stroobants^1,2^, Filipe Elvas^1,2^

^1^Antwerp University Hospital (UZA), Department of Nuclear Medicine, Edegem, Belgium;

^2^Molecular Imaging and Radiology (MIRA), University of Antwerp, Wilrijk, Belgium; ^3^Molecular Targeting Technologies, Inc., West Chester, PA, USA.

*These authors contributed equally for this work.

**Corresponding Author:** Filipe Elvas, Molecular Imaging Center Antwerp, University of Antwerp, Universiteitsplein 1, 2610 Wilrijk, Belgium.

Email: [filipe.elvas@uantwerpen.be](mailto:filipe.elvas@uantwerpen.be)

Telephone number: +3232654490

**Supplemental tables**

**Table S1.** [^99m^Tc]Tc-Duramycin release specification and validation run batch analysis.

| Test | Specification | Batch 1 | Batch 2 | Batch 3 |
| --- | --- | --- | --- | --- |
| 1. Total radioactivity | 740-1110 Mbq/batch | 814 | 930 | 751 |
| 1. Radionuclidic identity – gamma spectrometry | Gamma energy is 139 - 142 keV | 140.51 | 140.51 | 140.54 |
| 1. Radionuclidic purity | Max. 0.1 % ^99^Mo | No ^99^Mo detected | No ^99^Mo detected | No ^99^Mo detected |
| 1. Radiochemical purity | > 90% | 95.46 | 95.58 | 91.37 |
| 1. Radiochemical identity | Retention time: 15 – 16,5 min | 15.25 | 15.45 | 15.73 |
| 1. Appearance | Clear, free of particles | OK | OK | OK |
| 1. pH | pH of the finished product is 4.5-8.5 | 5 | 6 | 7 |
| 1. Ethanol concentration | ≤ 10% v/v | 9.8644 | 7.695 | 7.2465 |
| 1. Membrane filter integrity test | Bubble point > 3.2 bar | 3.3 | 3.5 | 4.1 |
| 1. Sterility | No growth of microorganisms after incubation at 37°C conform Ph. Eur. | No growth | No growth | No growth |
| 1. Bacterial endotoxins | < 17.5 IU/dose  (max. 16 ml) | < 2.57 IU/ml | < 2.50 IU/ml | < 2.50 IU/ml |

**Table S2.** Effective half-lives and residence times for all healthy volunteers and all regions of interest that were considered for the dosimetry calculations using the planar images.

| Region-of-interest | Volunteer 1 | | Volunteer 2 | | Volunteer 3 | | Volunteer 4 | | Volunteer 5 | | Volunteer 6 | | Mean | |
| --- | --- | --- | --- | --- | --- | --- | --- | --- | --- | --- | --- | --- | --- | --- |
|  | T_1/2_ | Ã/A_inj_ | T_1/2_ | Ã/A_inj_ | T_1/2_ | Ã/A_inj_ | T_1/2_ | Ã/A_inj_ | T_1/2_ | Ã/A_inj_ | T_1/2_ | Ã/A_inj_ | T_1/2_ | Ã/A_inj_ |
|  | [h] | [h] | [h] | [h] | [h] | [h] | [h] | [h] | [h] | [h] | [h] | [h] | [h] | [h] |
| Blood | 2.48 | 3.33 | 1.49 | 2.01 | 1.85 | 2.67 | 2.45 | 3.21 | 2.17 | 2.63 | 2.00 | 2.69 | 2.07 | 2.75 |
| Brain | 3.61 | 0.18 | 3.41 | 0.02 | 3.26 | 0.09 | 4.16 | 0.13 | 3.54 | 0.11 | 3.51 | 0.09 | 3.58 | 0.10 |
| Heart | 4.16 | 0.05 | 2.44 | 0.10 | 3.80 | 0.52 | 2.85 | 0.45 | 3.56 | 0.51 | 3.20 | 0.60 | 3.34 | 0.37 |
| Kidneys | 7.15 | 0.65 | 5.72 | 0.95 | 6.90 | 0.92 | 5.60 | 1.13 | 9.29 | 1.12 | 8.49 | 1.05 | 7.19 | 0.97 |
| Liver | 3.40 | 0.90 | 2.15 | 0.43 | 2.57 | 0.85 | 5.08 | 1.00 | 4.29 | 0.88 | 3.73 | 0.81 | 3.54 | 0.81 |
| Lungs | 3.61 | 0.35 | 2.99 | 0.37 | 3.15 | 0.33 | 3.73 | 0.52 | 3.43 | 0.29 | 3.09 | 0.28 | 3.33 | 0.36 |
| Spleen | 4.40 | 0.05 | 45.80 | 0.07 | 49.40 | 0.11 | 3.83 | 0.07 | 2.69 | 0.09 | 3.47 | 0.08 | 18.26 | 0.08 |
| Total body | 4.42 | 6.59 | 3.50 | 5.49 | 4.23 | 6.21 | 4.74 | 6.96 | 4.24 | 6.39 | 3.99 | 6.27 | 4.19 | 6.32 |
| Urinary bladder | 3.84 | 0.12 | 3.07 | 0.45 | 7.13 | 0.75 | 1.43 | 0.14 | 0.98 | 0.19 | 3.49 | 0.49 | 3.32 | 0.35 |

**Table S3.** Effective half-lives and residence times for all volunteers calculated based on the volumes-of-interest in the SPECT field-of-view.

| Volume-of-interest | Volunteer 1 | | Volunteer 2 | | Volunteer 3 | | Volunteer 4 | | Volunteer 5 | | Volunteer 6 | | Mean | |
| --- | --- | --- | --- | --- | --- | --- | --- | --- | --- | --- | --- | --- | --- | --- |
|  | T_1/2_ | Ã/A_inj_ | T_1/2_ | Ã/A_inj_ | T_1/2_ | Ã/A_inj_ | T_1/2_ | Ã/A_inj_ | T_1/2_ | Ã/A_inj_ | T_1/2_ | Ã/A_inj_ | T_1/2_ | Ã/A_inj_ |
|  | [h] | [h] | [h] | [h] | [h] | [h] | [h] | [h] | [h] | [h] | [h] | [h] | [h] | [h] |
| Kidneys | 9.09 | 0.28 | 9.01 | 0.45 | 9.34 | 0.42 | 8.34 | 0.27 | 8.49 | 0.35 | 6.20 | 0.28 | 8.41 | 0.34 |
| Liver | 4.98 | 1.04 | 3.35 | 0.45 | 3.38 | 0.84 | 5.73 | 0.83 | 4.66 | 0.62 | 4.40 | 0.59 | 4.42 | 0.73 |
| Spleen | 3.34 | 0.07 | 3.45 | 0.03 | 2.46 | 0.04 | 3.74 | 0.07 | 3.65 | 0.09 | 6.58 | 0.15 | 3.87 | 0.08 |

**Table S4.** Plasma protein binding expressed as % free radiotracer.

| Time pi | 5 min | 60 min | 180 min | 360 min | 1440 min |
| --- | --- | --- | --- | --- | --- |
| Volunteer 1 | 2.7 | 2.6 | 2.6 | 2.8 | 1.5 |
| Volunteer 2 | 3.8 | 3.1 | 2.7 | 2.4 | 3.0 |
| Volunteer 3 | 2.0 | 2.2 | 1.9 | 1.4 | ND |
| Volunteer 4 | 2.5 | 2.2 | 1.7 | 1.8 | ND |
| Volunteer 5 | 6.3 | 5.3 | 4.7 | 4.1 | ND |
| Volunteer 6 | 2.7 | 2.6 | 2.6 | 2.8 | ND |
